# The Cats‐and‐Dogs test: A tool to identify visuoperceptual deficits in Parkinson's disease

**DOI:** 10.1002/mds.27176

**Published:** 2017-10-04

**Authors:** Rimona S. Weil, Katerina Pappa, Rachel N. Schade, Anette E. Schrag, Bahador Bahrami, Dietrich S. Schwarzkopf, Sebastian J Crutch, Aidan G. O'Keeffe, Huw R. Morris

**Affiliations:** ^1^ Department of Molecular Neuroscience University College London London UK; ^2^ Dementia Research Centre University College London London UK; ^3^ Institute of Cognitive Neuroscience University College London London UK; ^4^ Department of Clinical Neuroscience University College London London UK; ^5^ Department of Experimental Psychology, University College London, London, UK, and Institute of Cognitive Neuroscience University College London London UK; ^6^ School of Optometry & Vision Science, Faculty of Medical & Health Sciences, University of Auckland Auckland New Zealand; ^7^ Department of Statistical Science University College London London UK

**Keywords:** vision, Parkinson's disease, cognition, dementia, biomarker

There are no robust features to predict which patients with Parkinson's disease (PD) will develop dementia. Those with involvement of visual processing regions are at highest risk of dementia.^1^, ^2^, ^3^ However, current measures of visuoperception are poorly sensitive.^4^ We have developed a sensitive test of visuoperception based on the clinical observation that patients with PD have difficulty reading distorted CAPTCHA (completely automated public Turing test to tell computers and humans apart) images.^5^

## Methods

### Participants

Twenty patients with PD and 11 age‐matched controls without eye disease or dementia were recruited. Clinical and detailed neuropsychological assessment was performed (Supplemental Table 1). Participants gave written informed consent. The study was approved by the local Research Ethics Committee.

### Procedure

**Figure 1:**
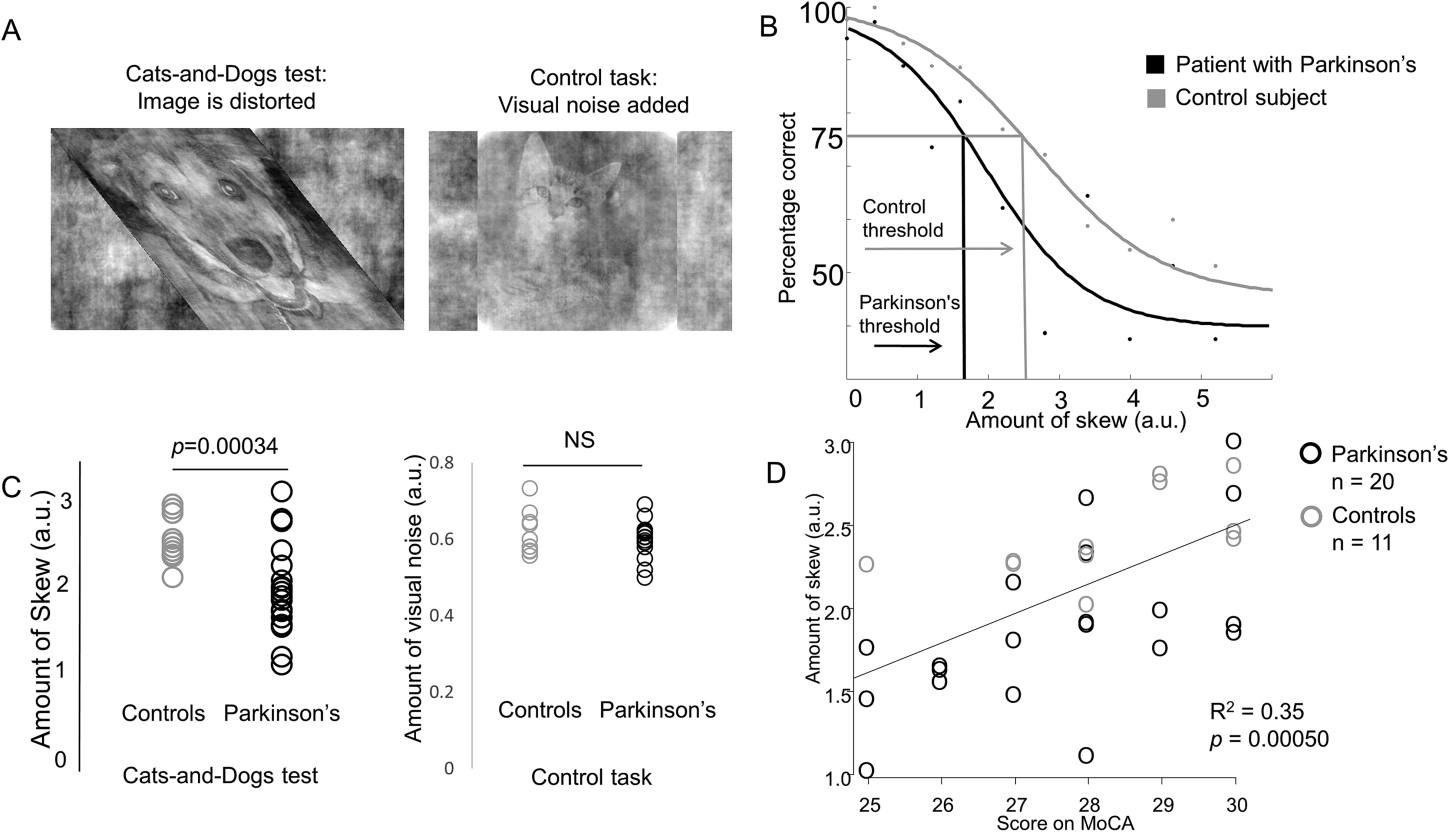
(**A**) Left: Cats‐and‐Dogs test, example skewed image. A dog is shown. Images in this task varied in the amount of skew, and performance at each level of skew was recorded. Right: control task, example image with added visual noise. A cat is shown. Images in this task varied in the amount of visual noise, and performance at each level of noise was recorded. (**B**) Method for determining performance in the Cats‐and‐Dogs test; psychophysical curves for 2 example participants are shown, one with PD (black), one without (gray). Percentage correct is shown for each level of skew. Performance is defined as the skew level corresponding to 75% (midway between perfect 100% and guess at 50%) and is marked for each of the participants. The same method was used to determine performance in the control task, with amount of noise plotted against percentage correct. (arbitrary units [a.u.]). (**C**) Left: performance in the Cats‐and‐Dogs test in patients with Parkinson's disease and controls. Patients with Parkinson's disease performed worse than healthy controls, with lower thresholds to correctly identify skewed images. Wider variation in performance was also seen in patients with Parkinson's disease. Right: performance in the control test in patients with Parkinson's disease and controls. There was no significant difference in performance in this task between patients with Parkinson's disease and healthy controls. (a.u.). (**D**) Relationship between performance in the Cats‐and‐Dogs test and overall cognition (Montreal Cognitive Assessment).

Images of cats and dogs were skewed by a variable amount (11 levels, 0‐5 arbitrary units [a.u.]) and combined with white noise (Fig. 1A and Supplementary Methods). On each trial the skewed image was shown for 280 milliseconds. Participants indicated whether the image was a cat or dog using the keypad (4 runs, each with 100 trials; 25 minutes in total, preceded by a practice session).

### Control Task

Images were prepared as above (but not skewed) with a varying proportion of visual noise added (11 levels), in a similar procedure with 2 runs, 100 trials per run, 15 PD patients and 10 age‐matched controls (Fig. 1A; Supplemental Table 2).

### Analysis

Demographic and neuropsychological data were compared using Welch's *t* and chi‐square tests. For each participant a psychophysical curve was generated for the Cats‐and‐Dogs test and control task, a sigmoid curve fitted, and 75% performance threshold determined (Fig. 1B). Bonferroni‐corrected *P* < 0.05 was considered significant. Linear regression was used to examine relationships between the Cats‐and‐Dogs test and other variables. We used a recently described algorithm, modified to include available clinical variables, to calculate each participant's risk of dementia. This combines cross‐sectional data including age, Movement Disorder Society Unified Parkinson's Disease Rating Scale (MDS‐UPDRS) motor score, depression, and REM‐sleep behavior scores to calculate the 2‐year risk of cognitive impairment.^6^

## Results

Patients with PD performed worse than controls at identifying skewed images: PD mean threshold, 1.92 ± 0.5 a.u; controls, 2.48 ± 0.26 a.u.; *t*_29.0_ = −4.06, *P* = 0.00034 (Fig. 1C). There was no other significant difference in cognitive or clinical tests, including the standard visuoperceptual tests, between PD patients and controls (excluding MDS‐UPDRS; Supplemental Table 1). Mean reaction times and visual acuity did not differ significantly between the groups.

There was no difference in the control task (white noise) between PD patients and controls.

The Cats‐and‐Dogs test correlated with higher age (estimate, −0.033 ± 0.009; *P* = 0.00093) and vascular risk, after adjustment for PD (estimate, −0.026 ± 0.006; *P* = 0.021). Even after age adjustment, it correlated with overall cognitive performance (estimate, 0.17 ± 0.05; *P* = 0.0037) and language, assessed with the Graded Naming Test (estimate, 0.076 ± 0.017; *P* = 0.00015; Fig. 1D) but not standard visuoperceptual tests (Supplemental Table 5). It also correlated with 2‐year cognitive impairment risk, calculated using cross‐sectional data^6^ (*R* ^2^ = 0.22, *P* = 0.0078; Supplemental Fig. 1).

## Discussion

We present pilot data suggesting that identifying skewed images in the Cats‐and‐Dogs test is a sensitive measure of visuoperception in early‐stage PD, with greater sensitivity than standard cognitive and visuospatial tests.

Performance on the Cats‐and‐Dogs test correlated with age, vascular risk, and cognitive performance but not with standard visuoperception tests, most likely because participants were at ceiling in these tests. Performance in this test also correlated with a prediction score for cognitive impairment in PD, suggesting that it may have utility as an early marker of cognitive decline, consistent with the literature of PD patients with involvement of visual‐processing regions being at the highest risk of dementia.^1^, ^2^, ^3^ However, our study will require replication in larger cross‐sectional and longitudinal numbers.

## Supporting information

Additional Supporting Information may be found in the online version of this article at the publisher's website.

Supporting Information

Supporting Information

Supporting Information

Supporting Information

Supporting Information

Supporting Information

Supporting Information

Supporting Information
