# Supplementary figures and images for: The Cats‐and‐Dogs test: A tool to identify visuoperceptual deficits in Parkinson's disease
Source: Mov Disord. 2017 Oct 4;32(12):1789–90. doi: 10.1002/mds.27176 (PMC5765443; doi:10.1002/mds.27176)

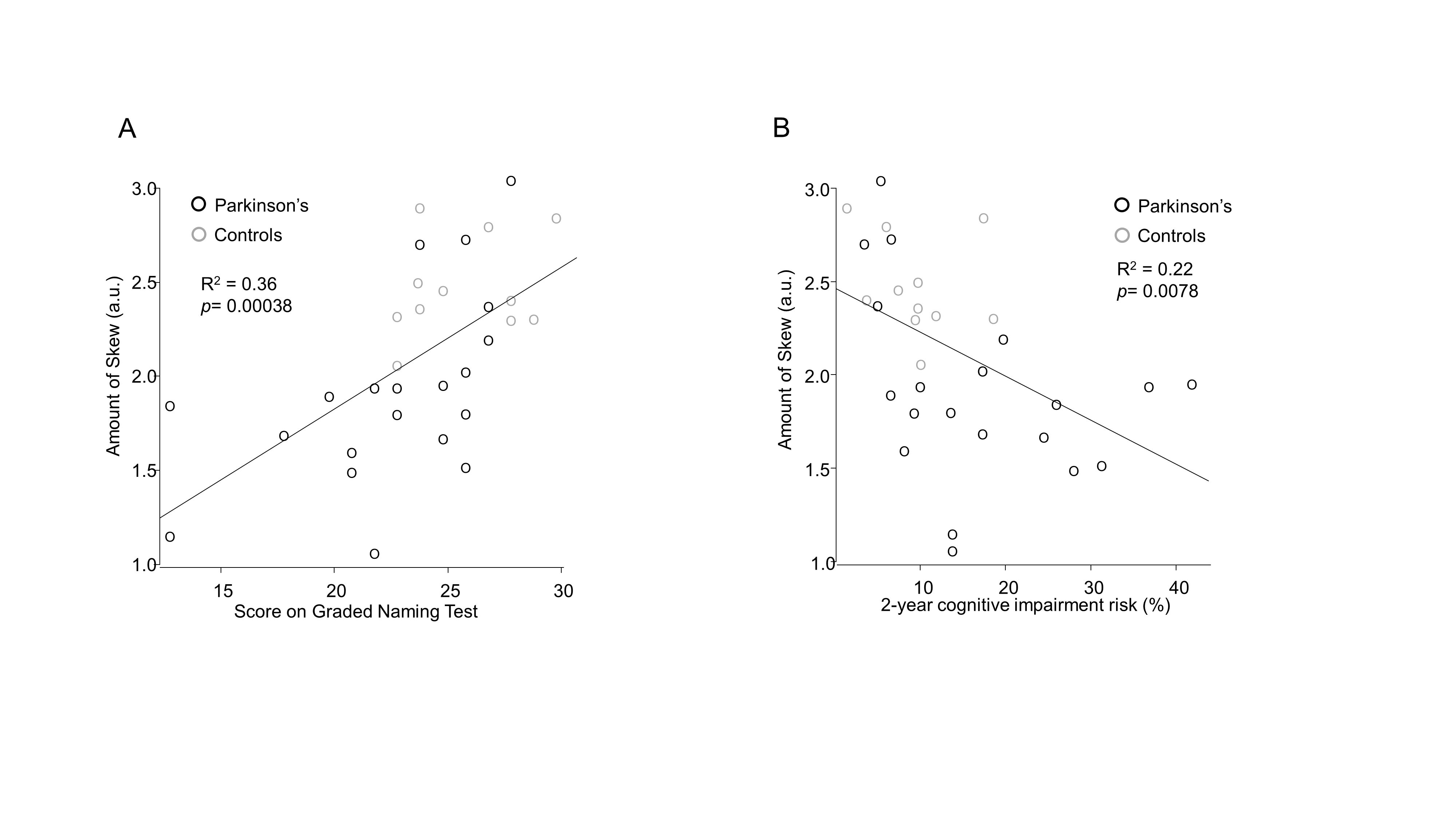

Supplement: Supplementary file 1 — Supporting Information [file MDS-32-1789-s001.tif]
