# Supplementary material for: The Cats‐and‐Dogs test: A tool to identify visuoperceptual deficits in Parkinson's disease
Source: Mov Disord. 2017 Oct 4;32(12):1789–90. doi: 10.1002/mds.27176 (PMC5765443; doi:10.1002/mds.27176)
Supplement: Supplementary file 2 — Supporting Information [file MDS-32-1789-s002.docx]

**Supplemental Table 1** Demographic details and clinical and cognitive performance of participants

|  | **Patients**  **n=20** | **Controls**  **n=11** | **t or** χ^2^ | ***p* value** |
| --- | --- | --- | --- | --- |
| Male / Female | 14/6 | 5/6 | 0.92 (1) | 0.18 |
| Age | 65.1(7.5) | 69(7.6) | -1.38 (20.6) | 0.18 |
| Disease duration | 3.61 (2.60) | - | - | NA |
| H&Y | 1.45 (0.60) | - | - | NA |
| UPDRS (total) | 38.7 (15.1) | 5.9 (4.3) | 9.0(24) | 3.5x10^-9^ ** |
| LEDD (mg) | 543.1 (354.4) | - | - | NA |
| Acuity | 0.76 (0.24) | 0.75 (0.23) | 0.06 (22.3) | 0.95 |
| **Cats-and-Dogs test** | **1.92 (0.5)** | **2.48 (0.26)** | **4.06 (29.0)** | **0.00034 **** |
| RBDSQ | 4.4 (3.1) | 2.1 (1.9) | 2.6 (28.7) | 0.015 |
| PDSS | 107.0 (18) | 121.6 (10) | -2.9 (28.9) | 0.0067 |
| HADS | 7.0 (3.2) | 5.9 (3.9) | 0.78 (17.5) | 0.45 |
| Vascular risk | 17.2 (11.3) | 18.1 (9.5) | -0.22 (23.9) | 0.83 |
| VFQ25 | 0.92 (0.09) | 0.93 (0.05) | -0.37 (24.3) | 0.71 |
| MoCA | 27.6 (1.7) | 28.3 (1.6) | -1.1 (22.7) | 0.28 |
| MMSE | 29.4 (1.2) | 29.8 (0.4) | -1.4 (25.5) | 0.17 |
| VOSP number location | 9.1 (1.3) | 9.5 (0.7) | -1.2 (29.0) | 0.26 |
| VOSP cubes | 9.4 (1.0) | 9.4 (0.8) | -0.12 (25.9) | 0.91 |
| VOSP fragmented letters | 18.8 (2.4) | 19.4 (0.9) | -0.9 (25.2) | 0.37 |
| VOSP Silhouettes | 19.6 (3.7) | 20.9 (4.3) | -0.88 (18.0) | 0.39 |
| VOSP Progressive silhouettes | 9.4 (2.5) | 10.1 (2.4) | -0.76 (21.4) | 0.46 |
| VOSP Object decision | 17.5 (1.8) | 18.4 (1.4) | -1.45 (25.6) | 0.16 |
| CORVIST shape discrimination | 7.8 (0.9) | 7.9 (0.3) | -0.35 (25.5) | 0.72 |
| CORVIST size discrimination | 1.8 (0.4) | 1.8 (0.6) | -0.096 (15.3) | 0.92 |
| CORVIST shape detection | 7.6 (1.0) | 7.6 (0.9) | 0.014 (22.5) | 0.99 |
| Letter fluency | 14.5 (4.9) | 17.2 (2.9) | -1.95 (28.7) | 0.061 |
| Category fluency | 21.2 (5.4) | 25.3 (5.6) | -1.91 (17.5) | 0.072 |
| Stroop (ink colour) | 68.3 (18.1) | 61.1 (13.5) | 1.27 (26.1) | 0.22 |
| Trails B-A | 46.9 (39.4) | 37.0 (10.2) | 1.06 (23.2) | 0.30 |
| RMT (words) | 45.5 (3.7) | 47.8 (1.9) | -2.27 (28.9) | 0.031 |
| GNT | 22.8 (4.3) | 25.9 (2.6) | -2.53 (28.7) | 0.017 |

Mean (SD) shown, except gender where actual numbers are shown. CORVIST, cortical vision screening test; GNT, graded naming test; HADS, hospital anxiety and depression scale; LEDD, Levodopa equivalent daily dose; MMSE, Mini Mental State Examination; MoCA, Montreal cognitive assessment; PDSS, Parkinson’s disease sleep scale; RBDSQ, REM sleep behaviour disorder screening questionnaire; RMT, recognition memory test (words); VFQ25, Visual function questionnaire; VOSP, visual object and space perception battery.

** Significant after correction for multiple comparisons.
