# Supplementary material for: The Cats‐and‐Dogs test: A tool to identify visuoperceptual deficits in Parkinson's disease
Source: Mov Disord. 2017 Oct 4;32(12):1789–90. doi: 10.1002/mds.27176 (PMC5765443; doi:10.1002/mds.27176)
Supplement: Supplementary file 3 — Supporting Information [file MDS-32-1789-s003.docx]

**Supplemental Table 2: demographics of participants in control task*.**

|  | **Patients**  **n=15** | **Controls**  **n=10** | **t or** χ^2^ | ***p* value** |
| --- | --- | --- | --- | --- |
| Male / Female | 11/4 | 3/7 | 4.6 | 0.03 |
| Age | 61.9 (6.1) | 61.3 (9.6) | 0.2 | 0.86 |
| Disease duration | 4.6 (2.3) | NA | NA | NA |
| H&Y | 1.64 (0.5) | 0 (0) | 12 | 1.510^-8^ |
| MDS-UPDRS (total) | 31.5 (13) | 3.4 (2.6) | 8 | 5.4x10^-7^ |
| Acuity | 1.08 (0.2) | 1.07 (0.17) | 0.16 | 0.87 |
| MoCA | 27.9 (2.1) | 29.2 (0.8) | -2.2 | 0.040 |

* 4 patients with PD and 2 controls took part in both the Cats-and-Dogs task and also the control task.

H&Y, Hoehn and Yahr; MDS-UPDRS, Movement Disorder Society Unified Parkinson’s Disease Rating Scale; MoCA, Montreal Cognitive Assessment;
