# Supplementary material for: The Cats‐and‐Dogs test: A tool to identify visuoperceptual deficits in Parkinson's disease
Source: Mov Disord. 2017 Oct 4;32(12):1789–90. doi: 10.1002/mds.27176 (PMC5765443; doi:10.1002/mds.27176)
Supplement: Supplementary file 4 — Supporting Information [file MDS-32-1789-s004.docx]

**Supplemental Table 3: missing data**

Number of subjects with data missing for each component of neuropsychology

| **Neuropsychology test** | **Number of subjects with missing data** |
| --- | --- |
| VOSP (all sections) | 2 |
| Subsection of VOSP | 2 |
| Category fluency | 1 |
| Stroop | 5 |
| Trails B-A | 5 |
| Recognition memory test (words) | 5 |
| Graded naming test | 1 |

VOSP, Vision Object and Space Battery;
