# Supplementary material for: The Cats‐and‐Dogs test: A tool to identify visuoperceptual deficits in Parkinson's disease
Source: Mov Disord. 2017 Oct 4;32(12):1789–90. doi: 10.1002/mds.27176 (PMC5765443; doi:10.1002/mds.27176)
Supplement: Supplementary file 5 — Supporting Information [file MDS-32-1789-s005.docx]

**Supplemental Table 4**

**Relationship between Cats-and-Dogs test and other disease factors**

Adjusted for presence of PD

| **Factor** | **R^2^** | **Estimate (SE)** | ***p* value** | **Estimate (SE)** | ***p* value:**  **adjusted for PD** |
| --- | --- | --- | --- | --- | --- |
| **Age** | **0.11** | **-0.022 (0.01)** | **0.065** | **-0.033 (0.009)** | **0.00093**** |
| Gender | 0.16 | 0.41 (0.2) | 0.025 | 0.29 (0.2) | 0.074 |
| Side affected^†^ (left, right or both) | 0.032 | -0.12 (0.2) | 0.45 | na | na |
| Tremor predominant^†^ | 0.020 | -0.14 (0.2) | 0.55 | na | na |
| Visual acuity | 0.0045 | 0.15 (0.4) | 0.72 | 0.16 (0.3) | 0.64 |
| **Vascular risk** | **0.26** | **-0.025 (0.008)** | **0.0031** | **-0.026 (0.006)** | **0.00017**** |
| RBDSQ | 0.052 | -0.039 (0.03) | 0.22 | -0.0043 (0.03) | 0.89 |
| PDSS | 0.012 | 0.0033 (0.006) | 0.56 | -0.004 (0.005) | 0.42 |
| HADS | 0.0013 | 0.0052 (0.03) | 0.85 | 0.017 (0.02) | 0.46 |
| Disease duration^†^ | 0.00090 | -0.0058 (0.045) | 0.90 | na | na |
| LEDD^†^ | 0.00044 | -3.0x10^-5^ (3x10^-4^) | 0.93 | na | na |
| UPDRS1 | 0.098 | -0.036 (0.2) | 0.086 | -0.0058 (0.02) | 0.79 |
| UPDRS2 | 0.16 | -0.038 (0.02) | 0.027 | -0.0031 (0.02) | 0.89 |
| UPDRS3 | 0.31 | -0.022 (0.006) | 0.0012 | -0.014 (0.009) | 0.14 |
| Age at diagnosis^†^ | 0.35 | -0.035 (0.01) | 0.0063 | na | na |
| **MoCA** | **0.35** | **0.18 (0.05)** | **0.00050**** | **0.15 (0.4)** | **0.00071**** |

HADS, hospital anxiety and depression scale; LEDD, Levodopa equivalent daily dose; MoCA, Montreal cognitive assessment; na, not applicable; PDSS, Parkinson’s disease sleep scale; RBDSQ, REM sleep behaviour disorder screening questionnaire; SE, standard error; UPDRS, Unified Parkinson’s disease rating scale (section 1 relates to non-motor symptoms, section 2 is a subjective assessment of movement, section 3 is objective movement assessment.

† In patients with PD only, therefore data not adjusted for PD.

** Significant after correction for multiple comparisons.
