# Supplementary material for: The Cats‐and‐Dogs test: A tool to identify visuoperceptual deficits in Parkinson's disease
Source: Mov Disord. 2017 Oct 4;32(12):1789–90. doi: 10.1002/mds.27176 (PMC5765443; doi:10.1002/mds.27176)
Supplement: Supplementary file 6 — Supporting Information [file MDS-32-1789-s006.docx]

**Supplemental Table 5**

**Relationship between performance in the Cats-and-Dogs test and performance in other cognitive domains**

Adjusted for age

| **Factor** | **R^2^** | **Estimate (SE)** | ***p* value** | **Estimate**  **(SE)** | ***p* value** |
| --- | --- | --- | --- | --- | --- |
| **MoCA** | **0.35** | **0.18 (0.05)** | **0.00050 **** | **0.17(0.05)** | **0.0037** |
| MMSE | 0.084 | 0.14 (0.08) | 0.11 | 0.11(0.09) | 0.23 |
| VOSP Number location | 0.10 | 0.15 (0.08) | 0.084 | 0.12(0.08) | 0.15 |
| VOSP cubes | 0.11 | 0.17 (0.09) | 0.074 | 0.12(0.1) | 0.26 |
| VOSP fragmented letters | 0.16 | 0.095 (0.04) | 0.028 | 0.09(0.04) | 0.033 |
| VOSP silhouettes | 0.0056 | 0.0097 (0.02) | 0.69 | 0.0063(0.02) | 0.78 |
| VOSP progressive silhouettes | 2.0x10-5 | 0.00090 (0.038) | 0.98 | 0.016(0.04) | 0.67 |
| VOSP object decision | 0.12 | 0.10 (0.05) | 0.058 | 0.090(0.05) | 0.085 |
| CORVIST shape discrimination | 0.013 | 0.078 (0.1) | 0.55 | 0.088(0.1) | 0.48 |
| CORVIST size | 0.12 | 0.38 (0.18) | 0.051 | 0.35(0.2) | 0.063 |
| CORVIST shape detection | 0.047 | 0.12 (0.1) | 0.24 | 0.085(0.09) | 0.38 |
| Letter fluency | 0.19 | 0.050 (0.019) | 0.013 | 0.051(0.02) | 0.0074 |
| Category fluency | 0.23 | 0.042 (0.01) | 0.0069 | 0.041(0.01) | 0.0064 |
| Stroop ink | 0.10 | -0.0096 (0.005) | 0.081 | -0.0081(0.005) | 0.13 |
| Trails B-A | 0.23 | -0.0074 (0.003) | 0.0067 | -0.0064(0.003) | 0.027 |
| RMT (words) | 0.27 | 0.078 (0.02) | 0.0030 ** | 0.069(0.025) | 0.011 |
| **GNT** | **0.36** | **0.076 (0.019)** | **0.00038 **** | **0.076(0.017)** | **0.00015** |

CORVIST, cortical vision screening test; GNT, graded naming test; HADS, hospital anxiety and depression scale; MMSE, Mini Mental State Examination; MoCA, Montreal cognitive assessment; PDSS, Parkinson’s disease sleep scale; RBDSQ, REM sleep behaviour disorder screening questionnaire; RMT, recognition memory test (words); SD, standard deviation; VOSP, visual object and space perception battery.

** Significant after correction for multiple comparisons.
