# Supplementary material for: The Cats‐and‐Dogs test: A tool to identify visuoperceptual deficits in Parkinson's disease
Source: Mov Disord. 2017 Oct 4;32(12):1789–90. doi: 10.1002/mds.27176 (PMC5765443; doi:10.1002/mds.27176)
Supplement: Supplementary file 7 — Supporting Information [file MDS-32-1789-s007.docx]

**Supplemental Table 6**

**Comparison of subdomains of MoCA between patients with PD and age-matched controls.**

| **Domain of MoCA (maximum possible score)** | **PD**  **Mean (SD)** | **Controls**  **Mean (SD)** | **t** | ***p*** |
| --- | --- | --- | --- | --- |
| **Visuospatial (5)**  **(trails, cube, clock drawing)** | 4.65 (0.49) | 4.91 (0.30) | -1.82 (28.5) | 0.079 |
| **Naming (3)** | 2.95 (0.22) | 2.91 (0.30) | 0.39 (16.2) | 0.70 |
| **Memory (delayed recall) (5)** | 3.65 (1.18) | 3.82 (1.66) | -0.30 (15.7) | 0.77 |
| **Attention (6)** | 5.75 (0.55) | 5.91 (0.30) | -1.0 (29.0) | 0.31 |
| **Language (3)** | 2.65 (0.67) | 2.73 (0.47) | -0.38 (27.2) | 0.71 |
| **Abstraction (2)** | 1.75 (0.55) | 1.82 (0.60) | -0.53 (19.1) | 0.76 |
| **Orientation (6)** | 6 (0) | 6 (0) | NA | NA (all subjects at ceiling) |
