# Supplementary material for: The Cats‐and‐Dogs test: A tool to identify visuoperceptual deficits in Parkinson's disease
Source: Mov Disord. 2017 Oct 4;32(12):1789–90. doi: 10.1002/mds.27176 (PMC5765443; doi:10.1002/mds.27176)
Supplement: Supplementary file 8 — Supporting Information [file MDS-32-1789-s008.docx]

**Supplemental Methods**

**Clinical assessment**

Symptoms were assessed using the MDS Unified Parkinson’s Disease Rating Scale (MDS-UPDRS)^1^. Predominance of tremor was calculated from the MDS-UPDRS motor score^2^. Visual acuity was measured using a 6m Snellen chart (with glasses) and converted to decimal acuity. Sleep was assessed using the Parkinson’s Disease Sleep Scale^3^ (PDSS) and the REM sleep Behavior Screening Questionnaire^4^ (RBDSQ); Mood was assessed using the Hospital Anxiety and Depression scale^5^ (HADS)). To enable the use of the cognitive risk algorithm, HADS scores were converted to Geriatric Depression Scores using a scalar conversion. Visual symptoms were assessed using the VFQ25 scale^6^. Vascular risk was estimated using the QRISK2 score (https://www.qrisk.org/2016/)^7;8^.

**Neuropsychology**

Participants underwent comprehensive neuropsychological assessment including general measures (mini-mental state examination^9^ (MMSE), Montreal Cognitive Assessment^10^ (MoCA) and specific cognitive domains including language (Graded Naming Test^11^), episodic memory (Recognition Memory Test for Words^12^) and visuo-perceptual and visuospatial functions (subtests of the VOSP^13;14^ (number location, cube analysis, incomplete letters, silhouettes, progressive silhouettes and object decision task), and subtests of the CORVIST (cortical vision screening test )^15^ (symbol acuity, shape discrimination, size discrimination and shape detection). Executive functions were assessed using the Trail Making Test (B-A)^16^, ink colour section of the Stroop (from the Delis-Kaplan Executive Functioning System)^17^ and with letter and category fluency subtests from the Wechsler Adult Intelligent Scale Revised^18^.

**Stimulus preparation:**

**Cats-and-Dogs test**

Images of cats and dogs were obtained from an open online database ([www.kaggle.com](http://www.kaggle.com)). 1000 images, comprising 500 cats and 500 dogs, were selected to show only one animal, centrally presented, with no overlapping objects. Cats and dogs were chosen as easily recognised and distinctive animals that nevertheless show similar low level characteristics. Images were cropped to be of equal size. For each run, 100 images were chosen and converted to grayscale. Fourier transforms of each image were computed, producing 100 magnitude and 100 phase matrices. The average magnitude matrix of all stimuli (cat and dog images) was stored for that run. On each trial, the phase matrix of a single cat or dog image was randomly selected and combined with a constant proportion (0.5) of a white noise matrix. This phase matrix was then recombined with the average magnitude matrix of the whole stimulus set using an inverse Fourier transform^19^. This process ensured that low-level image statistics were relatively constant across the stimulus set. The resulting image was then sheared by a variable amount of skew along the x-axis, using an affine matrix transformation, with amount of skew determined by a scalar in the transformation matrix. 11 levels of skew were used varying between 0 and 5 arbitrary units (a.u.), with the skew level chosen pseudo-randomly on every trial. The direction of skew was always towards the left side (see Fig. 1A). Pseudo-random presentation, rather than an adaptive staircase, was used, to avoid adaptation to the skew or gradual head tilt towards the direction of skew.

**Control task: visual noise as an alternative form of image distortion**

To test whether differences in identifying images are specific to skewing of the image, or might be found for other forms of image distortion, or simply be related to task difficulty, we tested a different form of distortion. Images were prepared as for the main study: For each run, 100 images were selected, converted to grayscale and Fourier transformed. On each trial, the phase matrix of a pseudo-randomly chosen image was recombined with the average magnitude matrix of that run. Images were not skewed, instead, a varying proportion of visual noise was added (11 levels of contrast between 0 and 1). The amount of noise varied pseudo-randomly on each trial. Visual noise was generated by producing a matrix of pseudo-random numbers drawn from the standard normal distribution, the same size as the image. This matrix was then superimposed onto the test image at varying levels of contrast (between 0 and 1) using the following formula:

Test image = Image * (1- Contrast level) + (Noise matrix * Contrast level)

Task and procedure for the control task was similar to the main task: 100 trials per run, 2-3 runs per participant. The control task was performed by 15 individuals with PD and 10 age matched controls, a subset of whom were the same individuals who had participated in the main experiment. (See Supplemental Table 2 for details of participants in the control task). The control task was performed at a separate testing session, as participants were unable to tolerate both tasks at the same session. Importantly, the control task therefore had similar properties to the main task, in terms of number of repetitions and timing of the stimulus.

**Supplemental Results**

**Impact of number of trial runs on performance**

As a post-hoc analysis, we repeated the analysis for the Cats-and-Dogs task, including only the first 2 runs, to ensure equivalence with the control task. We continued to see significant differences between patients with Parkinson’s and controls (p=0.003, t=-3.4). Thus, effects shown with the Cats-and-Dogs test are unlikely to be related to the number of trial repetitions.

**Comparison of the Cats-and-Dogs test with other measures of disease and cognition in PD**

Statistical differences between patients and controls were seen in sleep (both RBDSQ and PDSS) and for two measures of cognition: memory (RMT; *p*=0.031) and naming (GNT; *p*=0.017). After Bonferroni correction only the Cats-and-Dogs test showed significant differences between the groups. Of note, no differences were seen between the two groups in standard cortical vision tests and only one participant in the entire study made an error in copying pentagons, a commonly used test for visuo-perceptual performance^20;21^. No significant differences were seen between patients with PD and controls for subtests of the MoCA, including Clock Drawing (see Table 1 and Supplementary Table 6).

**Performance in Cats-and-Dogs test in Parkinson’s patients with mild cognitive impairment (MCI) and patients with normal cognition.**

Out of 20 patients with PD, 8 fulfilled level II criteria for MCI (40%)^22^, in line with previous estimates^23^. We found that Parkinson’s patients with MCI performed worse in the Cats-and-Dogs test (mean threshold 1.6 (±0.37)) compared with those patients with Parkinson’s and normal cognition (mean threshold (2.13 (±0.47), t(17.4)=-2.8, *p*=0.012). Patients with Parkinson’s and normal cognition also performed worse than controls (mean threshold 2.48 (±0.26), t(17.5) =-2.2, *p* =0.043).

**Figure Legends**

**Supplemental Fig. 1A.** Relationship between performance in the Cats-and-Dogs test and performance in a test of language (Graded Naming Test). (a.u. arbitrary units).

**Supplemental Fig. 1B.** Relationship between performance in the Cats-and-Dogs test and an independent predictor of 2-year risk of cognitive impairment in PD.

**Reference List**

**(1) Goetz CG, Tilley BC, Shaftman SR, Stebbins GT, Fahn S, Martinez-Martin P et al. Movement Disorder Society-sponsored revision of the Unified Parkinson's Disease Rating Scale (MDS-UPDRS): scale presentation and clinimetric testing results. *Mov Disord* 2008; 23(15):2129-2170.**

**(2) Stebbins GT, Goetz CG, Burn DJ, Jankovic J, Khoo TK, Tilley BC. How to identify tremor dominant and postural instability/gait difficulty groups with the movement disorder society unified Parkinson's disease rating scale: comparison with the unified Parkinson's disease rating scale. *Mov Disord* 2013; 28(5):668-670.**

**(3) Chaudhuri KR, Pal S, DiMarco A, Whately-Smith C, Bridgman K, Mathew R et al. The Parkinson's disease sleep scale: a new instrument for assessing sleep and nocturnal disability in Parkinson's disease. *J Neurol Neurosurg Psychiatry* 2002; 73(6):629-635.**

**(4) Stiasny-Kolster K, Mayer G, Schafer S, Moller JC, Heinzel-Gutenbrunner M, Oertel WH. The REM sleep behavior disorder screening questionnaire--a new diagnostic instrument. *Mov Disord* 2007; 22(16):2386-2393.**

**(5) Zigmond AS, Snaith RP. The hospital anxiety and depression scale. *Acta Psychiatr Scand* 1983; 67(6):361-370.**

**(6) Mangione CM, Lee PP, Gutierrez PR, Spritzer K, Berry S, Hays RD. Development of the 25-item National Eye Institute Visual Function Questionnaire. *Arch Ophthalmol* 2001; 119(7):1050-1058.**

**(7) Swallow DM, Lawton MA, Grosset KA, Malek N, Klein J, Baig F et al. Statins are underused in recent-onset Parkinson's disease with increased vascular risk: findings from the UK Tracking Parkinson's and Oxford Parkinson's Disease Centre (OPDC) discovery cohorts. *J Neurol Neurosurg Psychiatry* 2016; 87(11):1183-1190.**

**(8) Hippisley-Cox J, Coupland C, Vinogradova Y, Robson J, Minhas R, Sheikh A et al. Predicting cardiovascular risk in England and Wales: prospective derivation and validation of QRISK2. *BMJ* 2008; 336(7659):1475-1482.**

**(9) Folstein MF, Folstein SE, McHugh PR. "Mini-mental state". A practical method for grading the cognitive state of patients for the clinician. *J Psychiatr Res* 1975; 12(3):189-198.**

**(10) Nasreddine ZS, Phillips NA, Bedirian V, Charbonneau S, Whitehead V, Collin I et al. The Montreal Cognitive Assessment, MoCA: a brief screening tool for mild cognitive impairment. *J Am Geriatr Soc* 2005; 53(4):695-699.**

**(11) McKenna P, Warrington EK. Testing for nominal dysphasia. *J Neurol Neurosurg Psychiatry* 1980; 43(9):781-788.**

**(12) Warrington EK. Recognition Memory test: Manual. UKNFER-Nelson ed. Berkshire: 1984.**

**(13) Warrington EK, James M. The Visual Object and Space Perception Battery. Bury St Edmunds UK: Thames Valley Test Company; 1991.**

**(14) Rapport LJ, Millis SR, Bonello PJ. Validation of the Warrington theory of visual processing and the Visual Object and Space Perception Battery. *J Clin Exp Neuropsychol* 1998; 20(2):211-220.**

**(15) Plant GT, Warrington EK. The Cortical Vision Screening test (CORVIST). Bury St Edmonds: 2001.**

**(16) Reitan RM. A Manual for the Administering and Scoring of the Trail Making Test. 1959.**

**(17) Delis DC, Kaplan E, Kramer JH. Delis-Kaplan Executive Funciton System (D-KEFS) Examiner's Manual. San Antonio,TX: The Psychological Corporation; 2001.**

**(18) Wechsler D. Wechsler Memory Scale: Revised. 1987.**

**(19) Heekeren HR, Marrett S, Bandettini PA, Ungerleider LG. A general mechanism for perceptual decision-making in the human brain. *Nature* 2004; 431(7010):859-862.**

**(20) Williams-Gray CH, Evans JR, Goris A, Foltynie T, Ban M, Robbins TW et al. The distinct cognitive syndromes of Parkinson's disease: 5 year follow-up of the CamPaIGN cohort. *Brain* 2009; 132(Pt 11):2958-2969.**

**(21) Ala TA, Hughes LF, Kyrouac GA, Ghobrial MW, Elble RJ. Pentagon copying is more impaired in dementia with Lewy bodies than in Alzheimer's disease. *J Neurol Neurosurg Psychiatry* 2001; 70(4):483-488.**

**(22) Litvan I, Goldman JG, Troster AI, Schmand BA, Weintraub D, Petersen RC et al. Diagnostic criteria for mild cognitive impairment in Parkinson's disease: Movement Disorder Society Task Force guidelines. *Mov Disord* 2012; 27(3):349-356.**

**(23) Janvin CC, Larsen JP, Aarsland D, Hugdahl K. Subtypes of mild cognitive impairment in Parkinson's disease: progression to dementia. *Mov Disord* 2006; 21(9):1343-1349.**
